# Zukunft des agilen Lernens in der wissenschaftlichen Weiterbildung

**DOI:** 10.1007/978-3-662-62013-7_15

**Published:** 2020-07-15

**Authors:** Sandra Bräutigam, Florian Schindler

**Affiliations:** 1 SUSTAINUM – Institut für zukunftsfähiges Wirtschaften Berlin, Berlin, Germany; 2 Fraunhofer-Institut für Arbeitswirtschaft und Organisation IAO, Stuttgart, Germany; 3 ZNL TransferZentrum für Neurowissenschaften und Lernen der Universität Ulm, Ulm, Germany; 4 Fernstudieninstitut, Beuth Hochschule für Technik Berlin, Berlin, Germany; Beuth Hochschule für Technik Berlin, Fernstudieninstitut, Berlin, Deutschland

## Abstract

Es bedarf eines strukturell verankerten Prozesses, um durch lebenslanges Lernen die Mitarbeiter*innen in Unternehmen zu befähigen, mit den schnellen Zyklen der technologischen Entwicklungen Schritt zu halten. Dazu müssen die wissenschaftlichen Weiterbildungseinrichtungen an Hochschulen neue Angebote in ihr Portfolio aufnehmen. Daher lautet die Herausforderung im Sinne der von der Politik geforderten Durchlässigkeit von Hochschullehre und wissenschaftlicher Weiterbildung: Wie kann eine Angebotsdefinition und Weiterführung des agilen Lernens erfolgen? Was sind die Zukunftsaussichten des agilen Lernens als zu vermarktendes „Produkt“ im Hochschulkontext?

## Wissenschaftliche Weiterbildung im Wandel

Die digitale Transformation der Arbeitswelt stellt Unternehmen vor eine große Herausforderung. Um diese zu bewältigen, bedarf es für Mitarbeitende und Führungskräfte eines Erwerbs von neuen Kompetenzen. Diese Fähigkeiten und Kenntnisse sind nur zum Teil den digitalen Technologien an sich geschuldet. Vielmehr führen neue Strukturen in der Arbeitswelt zu einem erhöhten Bedarf an kollaborativen Fähigkeiten und kommunikativen Kompetenzen, aber auch zur Neudefinition von Team- und Führungsverhalten.

Die Frage, wie Menschen befähigt werden können, mit den schnellen Zyklen der technologischen Entwicklungen Schritt zu halten, rückt die Bedeutung von lebenslangem Lernen in den Fokus. Dazu „… müssen sich Aus- und Weiterbildung so schnell weiterentwickeln wie die moderne Arbeitswelt“ (Bundesbildungsministerin Anja Karliczek 2018). Derartige Entwicklungsaufgaben sind eindeutig an die Hochschulen und dort insbesondere an die wissenschaftlichen Weiterbildungseinrichtungen gerichtet. Eine Hochschule ist der tradierte Ort des Lehrens und Lernens mit wissenschaftlichen Methoden. Während an den Universitäten der Wissenschaftlichkeit von Methoden und Inhalten besondere Bedeutung im Hinblick auf eine Karriere in der Forschung zukommt, vermitteln die ehemaligen Fachhochschulen (University of Applied Sciences) Kompetenzen sehr praxisnah und anwendungsorientiert.

Betrachtet man das lebenslange Lernen als Schlüssel, um dem Wandel von Technologie und Gesellschaft durch Digitalisierung zu begegnen, sind die bisherigen Qualifizierungsformate von klar definierten Seminaren, Zertifikatskursen und Studiengängen nicht ausreichend flexibel. Daher stehen die Weiterbildungseinrichtungen von Hochschulen heute vor einer zentralen Herausforderung: Wie kann wissenschaftliche Weiterbildung als agiler Prozess realisiert werden, bei dem die Ziele eines individuellen Lernweges einem ständigen Wandel unterliegen und sich im Wechsel von Lern- und Arbeitsphasen weiterentwickeln? Die ehemaligen Fachhochschulen mit ihren jeweiligen Weiterbildungseinrichtungen sind hier besonders gefordert, neue Konzepte für Angebote an Unternehmen und Einzelpersonen zu entwickeln.

Damit ergeben sich neue Rollen für Anbieter sowie für Lehrende, die weiter durchdacht, erprobt und etabliert werden müssen. Der Fokus liegt dabei auf dem Einsatz von Lernprozessen, bei denen die Lernenden über das Lernen selbst mit anderen kommunizieren können und die Lernziele sich individuell formulieren und erarbeiten lassen. Das bedeutet, dass das Lernangebot viel stärker als bisher personalisiert, adaptiv (d. h. an das lernende Individuum angepasst) und kompetenzorientiert formuliert werden muss und die Möglichkeit, aus Fehlern zu lernen, im Prozess integriert sein sollte. Nur so wird diese „Weiterbildung 4.0“ auf Nutzbarkeit und Skalierung auch im Unternehmensalltag zugeschnitten.

Unter anderem am Fernstudieninstitut (FSI) der Beuth Hochschule für Technik Berlin wurde dazu in den Forschungsprojekten Brofessio und MeDiAL-4Q das *agile Lernen im Unternehmen* entwickelt und erprobt. Im Folgenden sollen exemplarisch am Beispiel dieser Weiterbildungseinrichtung FSI verschiedene Geschäftsmodelle für ein solches Lernangebot analysiert werden.

## Weiterbildung 4.0 am Fernstudieninstitut der Beuth Hochschule für Technik

Seit mehr als 35 Jahren bietet das Fernstudieninstitut (FSI) erfolgreich wissenschaftliche und berufliche Weiterbildung an. Im Fern- und Onlinestudium können Berufstätige zeitlich flexibel und ortsunabhängig einen zertifiziertern Weiterbildungsabschluss oder einen Masterabschluss (M.Eng., M.Sc.) erwerben. Enge Kooperationen mit Industrieunternehmen (wie z. B. Daimler AG, RENAC AG, Charité, Bayer AG, etc.) gewährleisten bereits eine große Praxisnähe und in Zusammenarbeit mit allen Fachbereichen der Hochschule wird für Kunden aus der Wirtschaft aktuelles Wissen durch externe Experten und Hochschullehrende vermittelt.

Das Fernstudieninstitut versteht sich als Serviceeinrichtung, da alle seine Angebote berufsbegleitend und kostenpflichtig und die Teilnehmenden damit automatisch entgeltpflichtige Kunden sind. Der Servicecharakter der Einrichtung sowie die hohe Kundenorientierung stellen auch den Geschäftsrahmen dar, denn das FSI ist gehalten, vollkostendeckend zu wirtschaften.

Der Ansatz des agilen Lernens wurde im Rahmen der Forschungskooperationen des Fernstudieninstituts mit der Fragestellung erforscht, inwieweit agiles Lernen in der wissenschaftlichen Weiterbildung im Hochschulkontext nutzbar gemacht werden kann. Das vielversprechendste Anwendungsszenario ist dabei, zertifizierbare agile Lernprojekte für Firmenkunden anzubieten.

Agiles Lernen, so wie es vom Fernstudieninstitut angewendet wird, bedeutet, dass der Lernprozess sich im Verlauf eines Lernprojekts an die Lernenden und an die Inhalte anpassen kann. Analog zum agilen Projektmanagement ist das „Endprodukt“ zu Beginn des Prozesses noch nicht detailliert festgelegt. Es gibt lediglich die Idee bestimmter Kernkompetenzen, die erworben werden sollen. Im Verlauf der dynamischen Entwicklung tauchen immer wieder neue Aspekte auf, die berücksichtigt werden müssen und die man zu Beginn des Entwicklungsprozesses noch nicht absehen konnte.

Für die Herausforderungen von modernen Unternehmen erscheint diese Methodik verglichen mit standardisierten Weiterbildungskursen als das sinnvollere Angebot. Über den Kompetenzerwerb hinaus unterstützt die agile Vorgehensweise Beschäftigte bei der Entwicklung von Problemlösungskompetenzen und stärkt so die Wettbewerbsfähigkeit der Unternehmen als lernende, sich weiterentwickelnde Organisationen – eine zentrale Voraussetzung für erfolgreiche Innovations- und Change-Prozesse.

Daher lautet die Herausforderung im Sinne der von der Politik geforderten Durchlässigkeit von Hochschullehre und wissenschaftlicher Weiterbildung: Wie kann eine Angebotsdefinition und Weiterführung des bisher entwickelten und erprobten agilen Lernens erfolgen? Was sind die Zukunftsaussichten des agilen Lernens als zu vermarktendes „Produkt“ im Hochschulkontext?

Konkret sind dafür folgende Fragen zu klären:

- Kann das agile Lernen zu einem regulären Hochschulangebot entwickelt und umgesetzt werden?
- Wie soll die Ansprachehaltung (Vermarktung) von agilem Lernen gegenüber Unternehmen als Bestandteil des Gesamtportfolios eines Hochschulinstituts realisiert werden?
- Wie hoch wäre der „Wert“ solcher Weiterbildungsmaßnahmen festzulegen im Spannungsfeld von Honorarordnungen und Kostenstellen einer öffentlichen Verwaltung?
- Wie ließe sich für derartige Angebote eine Zeitplanung realisieren, die unabhängig von Semesterabläufen durchführbar ist?

## Mögliche Integrationen des agilen Lernens in Hochschulstrukturen

Im Folgenden werden unterschiedliche Vorgehensweisen skizziert, die vom Fernstudieninstitut der Beuth Hochschule für Technik angedacht und zum Teil umgesetzt und weiterverfolgt wurden.

### Agiles Lernen als modernes Lernkonzept in bestehende Hochschul-Studienprogramme integrieren

Agiles Lernen kann als Methode im Unterricht bezogen auf ein bestimmtes Thema eingesetzt werden. Es ähnelt dabei dem Problem Based Learning (PBL). Von den Lernenden wird einerseits eine Eigenständigkeit in der Formulierung der Probleme wie auch der Lernziele erwartet, andererseits bedarf es in Anbetracht der vorgegebenen Semesterziele hierbei einer aufwändigen Hilfestellung durch die Lehrenden.

Vorteil: Insbesondere bei Angeboten für Erwerbstätige, die sich berufsbegleitend durch Zertifikatskurse oder ganze Masterstudiengänge weiterqualifizieren wollen, ist die Möglichkeit zum ortsunabhängigen und flexiblen „Blended Learning“ essenziell, d. h. die Kombination einer Vielzahl virtueller Tools und Methoden mit face-to-face Unterricht. Diese Kunden könnten vom agilen Lernen durch die weitere Flexibilisierung des Lernens sowie die Abfolge in kleineren Lernschritten und vor allem auch aufgrund der direkten Anwendbarkeit im eigenen beruflichen Umfeld profitieren.

Problem: Im agilen Lernen ist eine ergebnisoffene Vorgehensweise eine wichtige Voraussetzung. Im Hochschulkontext wird – auch in der Weiterbildung – von den Kunden aber bereits vor der Belegung eines Kurses oder eines Studiums die exakte Darlegung aller zu erwerbenden Inhalte sowie der zu erlangenden Kompetenzen gefordert. Der Nachweis dieser Kenntnisse und Kompetenzen wird auf Zertifikaten und Zeugnissen bei erfolgreicher Belegung ausgewiesen. Damit ist nur eine begrenze Ergebnisoffenheit möglich.

### Agiles Lernen als neues Produkt in das Weiterbildungsportfolio einer Hochschule integrieren

Das Angebot eines Kurses oder Moduls mit dem Titel „Agiles Lernen am Arbeitsplatz“ könnte sich an Unternehmen, Einzelpersonen oder andere Bildungseinrichtungen richten. Die Erweiterung des Angebots richtet sich dabei sinnvollerweise an bestehende oder potenzielle Kunden, z. B. produzierende Industrieunternehmen. Der thematische Inhalt des Angebots (z. B. Führungskompetenz) sollte vorgegeben sein, um den individuellen Betreuungsaufwand der Lehrenden nicht ausufern zu lassen.

Vorteil: Die Erweiterung des Angebots für Unternehmen durch ein individualisiertes, adaptives und dem Kundenproblem exakt angepasstes Lernkonzept. Ein darüber hinausreichender Marketingeffekt für etablierte Kurse wäre zu erwarten. Ein besonderer Anreiz für die HR Abteilungen der Unternehmen ist die Zertifizierbarkeit durch die Hochschule, wobei nach Bedarf sogar Credits und Workload ausgewiesen werden können.

Problem: Das Hindernis für ein solches Angebot liegt hierbei oft darin, dass die Weiterbildungseinrichtungen an den staatlichen Hochschulen in der Regel kein Mandat für einen beratenden Betrieb oder für ein Coaching von Unternehmen haben, sondern rein für die wissenschaftliche Weiterbildung als eine der Kernaufgaben staatlicher Hochschulen zuständig sind.

### Agiles Lernen als eigenständiges Produkt ausgliedern

Traut man dem agilen Lernen eine systemverändernde Kraft zu, kann es als eigenständiges Produkt angesehen werden. Das eigentliche Angebot an Unternehmens- oder Bildungseinrichtungs-Kunden könnte sowohl im thematischen Angebot (je nach Inhalt, Tiefe, Umfang) als auch im Methodensatz (Toolkit) verschiedene Ansätze umfassen.

Vorteil: Durch eine Eigenständigkeit des Angebots können Firmen als Kunden gezielt und individuell angesprochen werden. Agiles Lernen kann auf dem Markt platziert und sichtbar gemacht werden. Das Coaching bei den Unternehmen kann als Beratungsleistung oder als Projektleistung individuell und auftragsbezogen kalkuliert werden.

Problem: Für das Angebot muss als Organisationsform eine Ausgründung erfolgen. Entsprechende Förderprogramme wie beispielsweise EXIST sind auf Produkte mit hohem Anteil an Beratungsleistung nicht zugeschnitten. Eine finanzielle Förderung des Ausgründungsstarts ist daher schwierig. Ohne Anschubfinanzierung in einen Markt einzusteigen, in dem viele Konkurrenten sich ähnliche (weil ungeschützte) Labels geben, ist als finanzielle Herausforderung unattraktiv. Außerdem verliert die ausgegründete neue Organisation den Nachweis und die Kontinuität der in geförderten Drittmittelprojekten erarbeiteten Expertise.

### Agiles Lernen in neue Formate einbinden

Das duale Studium, sei es praxisintegriert oder auch ausbildungsintegriert verzeichnet für die Bachelorausbildung speziell in technischen Disziplinen eine zunehmende Nachfrage. Das Fernstudieninstitut der Beuth Hochschule für Technik achtet bei der Entwicklung dualer Studiengänge wie beispielsweise der Elektrotechnik oder der Mechatronik auf die iterative Abfolge von Lernsequenzen, bei denen vermitteltes Wissen immer wieder in der Praxis reflektiert wird. Dafür werden zunehmend auch kleine Lernteams gebildet, die selbstständig Problemlösungen erarbeiten.

Vorteil: Die besondere Flexibilität des agilen Lernens sowie die explizit gewinnbringende Anwendung auf die Herausforderungen am betrieblichen Arbeitsplatz machen es zu einem geeigneten Werkzeug für das zukunftsorientierte Lernen. Die parallelen Entwicklungen zukünftigen Lernens wie personalisiertes adaptives Lernen, flexibles Lernen, problemorientiertes Lernen (Problem Based Learning) und Lernen am Arbeitsplatz (Work Place Learning) verfolgen ähnliche Prinzipien wie das agile Lernen und sind damit anschlussfähig. Das macht agiles Lernen zum idealen Unterstützer neuer Bildungsformate, wie dem eingangs aufgezeigten dualen Studium.

Problem: Ein duales Studium richtet sich in erster Linie an Personen in der Erstausbildung und bietet für diese auch ein zukunfts- und praxisorientiertes Modell. Um dem Anspruch an ein Angebot für ein kontinuierliches, lebenslanges Lernen zu begegnen, kann die Integration des agilen Lernens in Duale Studienangebote nur ein erster Schritt sein.

### Agiles Lernen in eine bestehende oder eigenständige Gesellschaft auslagern

Befreit man das agile Lernen von der Struktur eines Kurses oder Moduls sowie von einer längerfristigen Planung, kann es als bedarfsorientierte Beratungs- oder Coaching-Leistung (z. B. für Unternehmen) angeboten werden.

Vorteil: Ein Unternehmenskonstrukt als GmbH oder gGmbH ließe sich als hundertprozentiges Tochterunternehmen der Hochschule betreiben. Hierfür gibt es gute Beispiele (WINGS der Universität Wismar, Humboldt Innovation GmbH, TUBS der Technischen Universität Berlin). Allerdings muss der Auftrag dieser Servicegesellschaften sehr klar von der Universität formuliert werden und auch das finanzielle Zusammenspiel muss im Grundsatz geklärt sein. Eine Variante dieses Geschäftsmodells ist die Auslagerung von aus der Forschung erwachsenen Produkten in ein bereits bestehendes hochschulexternes Unternehmen.

Problem: Das Problem bei solchen Auslagerungen oder Verwertungen ist die Definition von Schnittstellen und die Vermeidung von Subventionen für einzelne Unternehmen. Sollten einzelne Komponenten des agilen Lernens Nachfrage am Markt erfahren, so könnten natürlich Wirtschaftsunternehmen diese Komponenten vermarkten. Die Hochschulen müssten diese Komponenten fair und öffentlich anbieten und eine regelgerechte Vergabe an Wirtschaftsunternehmen sicherstellen.

## Agiles Lernen weiterentwickeln

Ein wesentlicher Aspekt der Weiterentwicklung sollte auf die „geographically dispersed teams“ ausgerichtet sein. Dabei handelt es sich um verteilte Teams, die mehr trennt als nur die Zeitverschiebung, weshalb zwingend auch eine kulturelle und soziale Diversität einbezogen werden muss. Das agile Lernen in virtuellen Gruppen kann und wird in Zukunft durch Augmented und Virtual Reality Technologien (AR/VR) erweitert werden.

Die zentralen Vorteile sowohl der Didaktik des agilen Lernens als auch der Möglichkeiten von AR/VR-Technologien greifen komplementierend ineinander. Mit dem Konzept der Lernkarten (siehe Abschn. 10.1007/978-3-662-62013-7_14#Sec3) gibt es bereits interaktive Selbstlernformate, die für agile Lernprojekte im Arbeitsprozess optimiert wurden. In Kombination mit einer intelligenten AR-Brille könnten in Zukunft beispielsweise für die Anwender Lernkarten gegenstandsbezogen eingeblendet werden, wobei diese gleichzeitig ihre Hände frei haben, um eine Tätigkeit direkt zu erproben. Der individuelle Lernprozess erfolgt damit adaptiv, problembezogen und anwendungsorientiert.

Gleichzeitig muss die Zusammenarbeit von verteilten Teams als der neue Normalfall verstanden werden und als zentrales Prozesselement in die Konzeption einfließen. Hierfür soll das agile Lernen für Arbeits- und Lernprojekte in virtuellen Teams erweitert und optimiert werden. Geeignete Anwendungsfälle ergeben sich durch das kollaborative Lernen und Arbeiten im Rahmen von Servicetrainings, die zwischen erfahrenen Fachkräften am Firmenstandort und Fachkräften an Außenstandorten stattfinden.

Durch Einbindung großer Konzerne und mittelständischer Firmen kann das Konzept des agilen Lernens bekannt gemacht werden, so dass reale und virtuelle Agile-Learning-Centers aufgebaut werden können. Die individuelle Komponente des agilen Lernens kann zum komplett personalisierten und adaptiven Konzept weiterentwickelt werden.

Hinsichtlich der unter 10.1007/978-3-662-62013-7_1#Sec2 aufgeworfenen Fragen muss zusammenfassend festgestellt werden, dass sich derzeit keines der unter 10.1007/978-3-662-62013-7_1#Sec3 entworfenen Szenarien realisieren lässt. Daran ist an Hochschulen und deren Weiterbildungseinrichtungen in der Zukunft weiter zu arbeiten. Gleichzeitig wurde durch die Coronavirus Pandemie an Hochschulen, Unternehmen und für Individuen ein nie dagewesener Prozess der Umstrukturierung und Umorientierung bezüglich digitaler Arbeit angeschoben. Die zu erwartende nachhaltige Veränderung etablierter Strukturen kann das agile Lernen zum Modell der Zukunft machen. Daraus lässt sich die Notwendigkeit ableiten, agiles Lernen schwerpunktmäßig zum praxisorientierten Lernen der digitalen Zukunft weiterzuentwickeln und im Portfolio der Weiterbildungseinrichtungen von Hochschulen durch entsprechende Vereinbarungen und Erweiterungen zu verankern.
